# A National Dental Society

**Published:** 1884-02

**Authors:** 


### A NATIONAL DENTAL SOCIETY.

There is much in the idea of one central association that shall be the recognized head of the profession in this country, that commends it to the consideration of every progressive man. England has such an one, and it is argued why should not we? Why cannot all the dentists of America unite and hand in their allegiance to such an organization? Much was expected in certain quarters from the National Association that was founded a few years since. That organization has not, however, fulfilled the hopes of its founders, and is already in a moribund condition. There were original elements in it that were prejudicial to its success. There was a suspicion of ulterior objects, and that there were dull personal axes to be ground to a fine edge. Besides, it was too comprehensive in its character, and too broad for practicability. A compact, easy working national society, is not as feasible in this country as in England, because of our great extent of territory, and the consequent diversity of interests and characteristics in the several great sections. Eastern men and western men, northern men and southern men, have certain sectional peculiarities that, in the transaction of the business affairs of such a society, do not easily harmonize. We may deprecate this, and assert that it should not be, but the plain facts of the case are that they do exist, and this must be taken into consideration whenever members of the whole profession are to be brought into association.

Then, again, climatic influences cannot be ignored. The northern dentist is most free to attend the necessarily lengthened sessions of a national society during his vacation, and that is in midsummer. He naturally seeks a place where the temperature is not too high, and no great number of them can be persuaded to attend a meeting held in the Southern States during that season. Southern men complain if the meetings be not held with them occasionally, and not unnaturally accuse their brethren of sectionalism when this is declined, and it is this climatic obstacle that has been the chief hinderance to the representation of all parts of the country in the American Dental Association. No society has ever, in this country, made such a record as has this, and to-day it seems to be as near the typical national association as it is at present practical to attain. But it is mainly made up of delegates and members from but a few of the States. The Pacific States are practically unrepresented, and it has but few members from the extreme south.

The Southern Dental Association is doing excellent work, and that organization should be sustained. There are enough of good men who will but occasionally visit the American Association to form an excellent society. Let the southern men then continue their meetings, and when a society shall have been formed upon the Pacific slope that shall quite cover that ground, the profession will be comparatively well organized, and if then the best men of each of the societies will, as convenience permits, attend the meetings of the others, fraternal intercourse will be kept up, and the general spread of professional intelligence will be assured.

The American Dental Association should retain its national character, and should be recognized as the representative head of dentistry in America, but it should not attempt too much. The country is too large to secure a general attendance upon the meetings of any one society. There is no reason why a Southern and a Pacific society should not become accepted representatives of the profession in each of those sections.

The founding of a National Museum at Washington, which shall be acknowledged by the government, and shall receive contributions, not only from the profession at large but from the army and navy of the country, is a thing to be earnestly desired. But such an institution need not be under the fostering care of any national society. Indeed, there are some strong reasons why it should, like the Medical Museum, be under the care of the general government, and be directly in charge of some government official. Its permanence would be thereby assured, and the co-operation of those who can best serve its interests would be secured. It might even be a branch of the medical department of the army and navy, but certainly the countenance and assistance of that bureau should be gained. We believe that steps have already been taken and a movement inaugurated which will, we hope, culminate in the success of an object fraught with so much of good to dentistry and to the world.

